# Automatic detection algorithm for establishing standard to identify “surge blood pressure”

**DOI:** 10.1007/s11517-020-02162-4

**Published:** 2020-04-13

**Authors:** Ayako Kokubo, Mitsuo Kuwabara, Hiroshi Nakajima, Naoko Tomitani, Shingo Yamashita, Toshikazu Shiga, Kazuomi Kario

**Affiliations:** 1 Development center, Technology Development HQ, Omron Healthcare Co., Ltd, 53 Kunotsubo, Terado-cho, Muko, Kyoto, 617-0002 Japan; 2 Division of Cardiovascular Medicine, Department of Medicine, Jichi Medical University School of Medicine, 3311-1, Yakushiji, Shimotsuke, Tochigi, 329-0948 Japan; 3 Technology and Intellectual Property H.Q., Omron Corporation, 9-1 Kizugawadai, Kizugawa-city, Kyoto, 619-0283 Japan

**Keywords:** Clinical informatics, Medical informatics applications, Expert systems, Learning from labeled data, Blood pressure monitors

## Abstract

Blood pressure (BP) variability is one of the important risk factors of cardiovascular disease (CVD). “Surge BP,” which represents short-term BP variability, is defined as pathological exaggerated BP increase capable of triggering cardiovascular events. Surge BP is effectively evaluated by our new BP monitoring device. To the best of our knowledge, we are the first to develop an algorithm for the automatic detection of surge BP from continuous “beat-by-beat” (BbB) BP measurements. It enables clinicians to save significant time identifying surge BP in big data from their patients’ continuous BbB BP measurements. A total of 94 subjects (74 males and 20 females) participated in our study to develop the surge BP detection algorithm, resulting in a total of 3272 surges collected from the study subjects. The surge BP detection algorithm is a simple classification model based on supervised learning which formulates shape of surge BP as detection rules. Surge BP identified with our algorithm was evaluated against surge BP manually labeled by experts with 5-fold cross validation. The recall and precision of the algorithm were 0.90 and 0.64, respectively. Processing time on each subject was 11.0 ± 4.7 s. Our algorithm is adequate for use in clinical practice and will be helpful in efforts to better understand this unique aspect of the onset of CVD.

**Electronic supplementary material:**

The online version of this article (10.1007/s11517-020-02162-4) contains supplementary material, which is available to authorized users.

**Graphical abstract:**
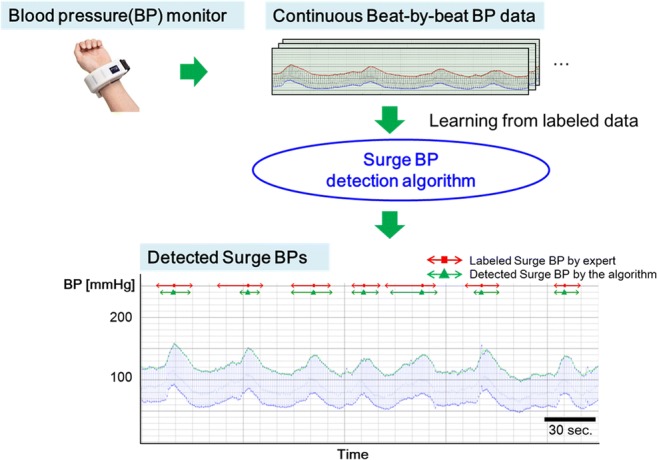
Surge blood pressure (surge BP) which is defined as pathological short-term (several tens of seconds) exaggerated BP increase capable of triggering cardiovascular events. We have already developed a wearable continuous beat-by-beat (bBb) BP monitoring device and observed surge BPs successfully in obstructive sleep apnea patients. In this, we developed an algorithm for the automatic detection of surge BP from continuous BbB BP measurements to save significant time identifying surge BP among > 30,000 BbB BP measurements. Our result shows this algorithm can correctly detect surge BPs with a recall of over 0.9.

## Introduction

Management of 24 h blood pressure (BP) is important for preventing the progress of hypertensive target organ damage and onset of cardiovascular disease (CVD) such as stroke or heart disease [1, 2]. As home BP monitoring (HBPM) devices are increasingly used by consumers, it has become easier to monitor and control morning and evening BP. However, even if they are controlled, there is still a risk of masked uncontrolled BP (daytime or nighttime). In particular, several studies have demonstrated that the mean value of nighttime BP measurements taken by ambulatory BP monitoring (ABPM) with fixed time intervals (e.g., 30 min.) is a stronger predictor of cardiovascular (CV) events than daytime ambulatory or clinic BP [3–6]. In addition, an increase in nighttime BP variability (BPV), defined as the standard deviation of nighttime BP measured by ABPM, is related to risks of CV events [2, 7]. It is assumed that some pathological factors such as obstructive sleep apnea (OSA) and microarousal increase nighttime BPV. It is known that these pathological factors generate short-term BPV [8–10]. Characteristic of this kind of BPV is a sharp rise in BP over several tens of seconds. We define pathological short-term exaggerated BP increase that can trigger a CV event as “surge BP” [1, 2, 11]. Although it is important to detect such surge BP for detailed assessment of CV risk, ABPM, which is the gold standard of nighttime BP measurement, cannot detect surge BPs precisely because its intermittent measurements (at 15–30-min intervals) would miss the surge BP peaks.

To resolve the unmet need to detect cases of surge BP during sleep apnea episodes in OSA patients, we developed hypoxia-trigger nocturnal HBPM and have studied the clinical implication and management of surge BPs in OSA patients [1, 2, 11–17]. However, as this HBPM is based on the oscillometric method, the peak of surge BP might be underestimated. Consequently, we developed a wearable continuous beat-by-beat (BbB) BP monitoring device using a tonometry method [18], which measures BP generated by each heartbeat. Using this device, we already have observed surge BPs successfully in OSA patients [1, 2, 11]. Further, we found that the peak of surge BP detected by our BbB BP monitoring device was higher than that detected by hypoxia-trigger BP monitoring [2, 11].

However, in severe OSA patients, several hundred surge BPs occur among > 30,000 BbB BP measurements recorded in a single night. It is too time-consuming for clinicians to review all the raw continuous BbB BP data manually to identify these surge BPs correctly. Even if a clinician spends a lot of time reviewing all the big data, the results of surge BP could vary widely from clinician to clinician because, as yet, there is no solid standard to identify surge BP. Furthermore, even if the same clinician reviews the entire dataset for a given patient, the clinician’s judgment is still subjective and might change or be in error depending on work environment or level of fatigue.

Thus, in this study, we developed a detection algorithm that automatically identifies surge BP by using fixed criteria from significant amounts of raw continuous BP data. We evaluated the performance of our algorithm by analyzing consistency between surge BP results detected by the algorithm and those identified by experts who have specialized knowledge about CV medicine.

## Materials and methods

We describe an overview of how the algorithm was developed. The requirements for the algorithm are presented in the “Requirement of the algorithm” subsection. Subsections and Beat-by-beat BP measurement” and “Collection of surge BP labeled by CV experts” describe the data collection procedures. These include labeling of surge BPs to train the algorithm. The constitution of the algorithm functions is provided in the “Development of the algorithm” subsection and the methods used to evaluate performance are given in the “Performance evaluation” subsection.

### Requirement of the algorithm

The standard criteria or algorithm of surge BP detection have not existed until now. Thus, our algorithm could be an objective and reliable method to identify surge BP. It also could encourage future clinical studies relating to surge BPs. Surge BPs detected by the algorithm could be used for summary reports, whereby clinicians could evaluate the results of overnight BbB BP measurement and discuss their assessment with their patients. Taking these situations into consideration, three types of requirements (described below) are needed for the algorithm:

1. High sensitivity of surge BP detection: To avoid underestimation of patients’ CV risks, surge BP should be detected with high sensitivity even if it includes undetermined BPV in terms of its shape or degree.
2. Understandability of detection rules: To be an acceptable algorithm for wide use among clinicians, the rule should be fully understandable [19]. Experimental rules for identifying surge BP are formulated as detection rules in the algorithm to gain acceptance of clinicians. Thus, surge BPs should be reviewed and manually labeled by CV experts. For explicability from a medical perspective, knowledge of CV medicine should also be applied when the algorithm works. The understandable detection rule is easily edited even if the criteria are necessary to change considering the result of the future clinical study, which reveals features of surge BP associated with CV events.
3. High processing speed: Because, as mentioned above, the results of surge BP detection will be used for reports in consultation with patients, it is necessary to generate a report in a relatively short time.

### Beat-by-beat BP measurement

#### Measurement device

**Fig. 1:**
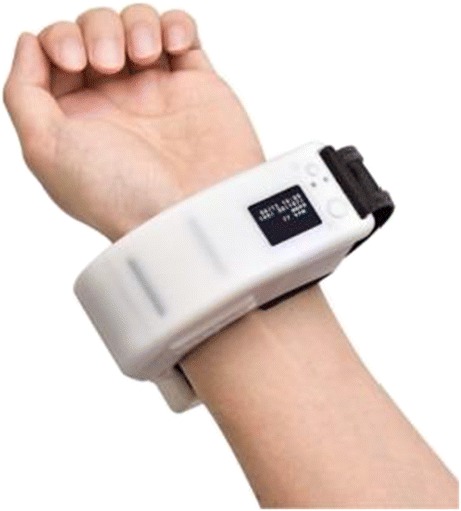
Beat-by-beat blood pressure monitoring device [1]

This study was conducted by using BbB BP monitoring device which we recently developed, which enables the measurement of pulse waves at a sampling rate of 125 Hz and calculates each BP from these pulse waves [1] (see Fig. 1). The device consists of a tonometry sensor and a BP measurement unit using the cuff oscillometric method which is widely used in automatic BP monitors. The tonometry sensor consists of an array of pressure sensors that are pressed against the skin over a radial artery. At the beginning of continuous BP measurement, the pressure measured by tonometry sensor must be calibrated by BP measured using the oscillometric method to output continuous absolute BP values. Recalibration is needed when contact between the skin and the tonometry sensor changes because of body motion. Our device automatically recalibrates when the device senses abnormal BP values.

#### Flow of study subject selection

**Fig. 2:**
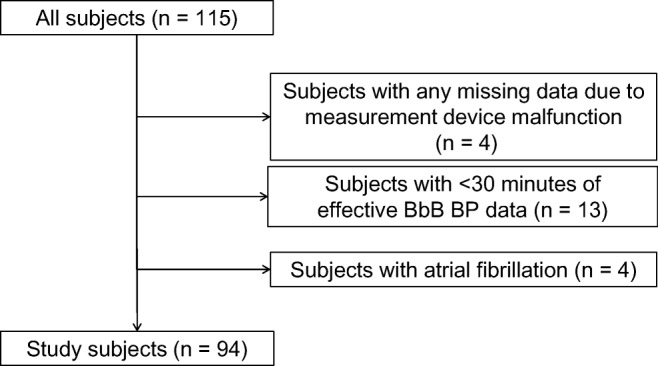
Flowchart of the study subject selection. A total of 115 subjects participated in this study and 94 subjects were included as the study subjects

Figure 2 shows the flowchart of the study subject selection. Initially, 115 subjects were recruited for the study. The inclusion criteria were an age of > 20 years and patients with a sleep disorder or nocturnal hypertension who are expected to have frequent surge BPs. The BbB BP monitoring device was worn on the subject’s left wrist in the supine position by the members of the study. The subjects were able to turn over in bed and go to the toilet. After wearing the device, they were requested to sleep overnight. The length of recordings was approximately 10 h, which depended on the time of the device setting and the patient’s wakeup time. This study was conducted at Washiya Hospital, Tochigi, Japan, from April 2016 to March 2018. The study was approved by the institutional research board of the Jichi Medical University School of Medicine (rin-dai15-117 and rin-B17-028), and all subjects provided their written informed consent to participate. After excluding 21 subjects as shown in Fig. 2, a total of 94 subjects were included as the study subjects. As shown in Fig. 2, subjects with missing data or with < 30 min of effective BbB BP data were excluded for their low reliability. Subjects with atrial fibrillation (AF) were also excluded, because their BbB BP data was not suitable as training data for developing the algorithm due to the large variability of the time interval between heart beats.

**Fig. 3:**
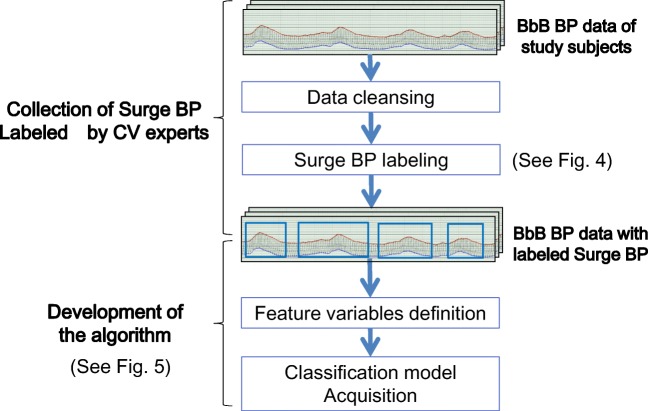
Overall structure for algorithm development. Collected BbB BP data is given labels of surge BP by cardiovascular experts. These surge BP labels are used for development of the algorithm

We developed the algorithm by using the BbB BP data obtained from these 94 study subjects as described in Fig. 3 and below, in the “Collection of surge BP labeled by CV experts” and “Development of the algorithm” sections.

### Collection of surge BP labeled by CV experts

**Fig. 4:**
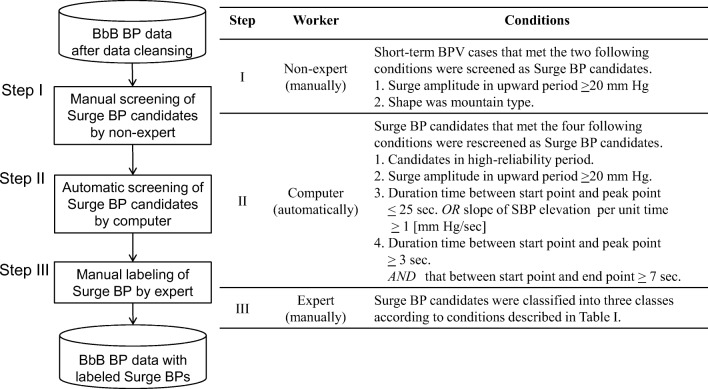
Steps in surge BP labeling. BPV: blood pressure variability, SBP: systolic blood pressure

Our algorithm was developed using surge BPs labeled by a CV expert using the following procedures. For data cleansing, we removed low-reliability measurement periods to ensure high-quality data for training the algorithm. The procedure is shown in the supplementary material section—Data cleansing. After data cleansing, only surge BPs observed during high-reliability periods were labeled to assure good quality training data. The labels include the start, peak, and end points. These “feature points” express the shape of the surge BP. Surge BPs were screened and labeled in accordance with the procedure and conditions as shown in Fig. 4.

In step I (Fig. 4), potential surge BPs that were in high-reliability periods were screened manually with high sensitivity by nonexpert study staffs. The staff members received training for > 1 week for surge BP labeling before they started the work. In step II, surge BP candidates screened in step I were subjected to screening refinement to reduce the impact of subjective error induced by a staff’s condition (e.g., fatigue or work environment). In step III, a CV expert visually checked the shape of surge BP candidates to improve its precision and classified them into three classes as: “surge BP,” undetermined BPV,” and “noisy BPV” based on Table 1. The undetermined BPV means that the BP varies certainly but neither surge BP nor the noise. The judgment criteria used in steps I, II, and III were reviewed and approved by a specialist clinician (Kario), who is a coordinator of the study and has specialized knowledge of CV medicine, and by an expert (Kuwabara) who has considerable experience in clinical research in CV medicine. Therefore, the developer of the algorithm (Kokubo) did not get involved in the surge BP labeling but instead relied entirely on these expert judgments.

**Table 1:** Conditions for surge BP classification

Class	Condition
Surge BP	Surge BP candidates that were not otherwise classified.
Undetermined BPV	Surge BP candidates that met any of the four following conditions by cardiovascular expert’s subjective judgment. 1. Slope of SBP elevation per unit time was small. 2. Variability of SBP in upward period was large. 3. Physiological BPV caused by respiration. 4. Surge BP candidates, including instantaneous BPV due to body motion.
Noisy BPV	Surge BP candidates that met either of the two following conditions by cardiovascular expert’s subjective judgment. 1. BPV caused by body motion. 2. Surge BP candidates that did not recover to the level of SBP at start point from that at peak point in downward period.

*BPV* blood pressure variability, *SBP* systolic blood pressure

### Development of the algorithm

**Fig. 5:**
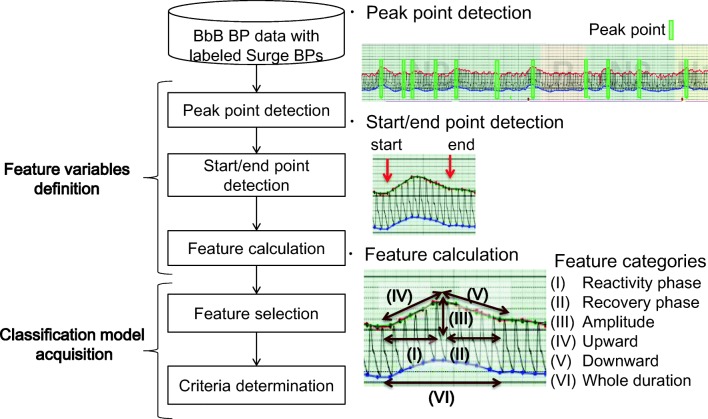
Development steps for surge BP detection algorithm. Feature variables representing surge BPs are defined and used for modeling classifier

We developed an algorithm that classifies surge BP candidates into “surge BP” or “not surge BP”. High detection sensitivity is required in the algorithm. However, detecting surge BP is challenging because of fluctuations in BbB BP and various surge BP patterns in terms of time and amplitude domains. We solved these problems considering the variable and complex property of surge BP and compose the algorithm of “feature variables definition” and “classification model acquisition” (see Fig. 5). In the feature variable definition block, the features of surge BP were defined using knowledge of CV medicine. The processing steps of feature variables definition were composed to gradually narrow down the surge BP candidates to realize highly sensitive detection. In classification model acquisition, features to be used for the classification were selected, and their criteria were determined using a supervised learning.

#### Feature variable definition

This block consists of “peak-point detection” and “start/end-point detection,” which are the preprocessing steps, and “feature calculation” (see Fig. 5). Here the preprocessing parts are described in the supplementary material section—Details of feature variable definition.

In feature calculation, six categories of surge BP features based on the surge BP shape were set (Fig. 5). They represent the clinician’s viewpoint: reactivity phase, recovery phase, amplitude, upward, downward, and whole duration. Then, we designed 48 features in those categories, including some features defined in a previous study [20], and used them as input variables in later feature selection. These categories and features were reviewed by experts to ensure meaningfulness of them in terms of CV medicine.

#### Classification model acquisition

This block consists of two steps: “feature selection” and “criteria determination” (see Fig. 5). The classifier was given as a function *f*, which is the product of function *g*(*x*_*i*_, *a*_*i*_), where *g* is a threshold function that determines whether values of the variable *x*_*i*_ exceed a given threshold *a*_*i*_. The output of *g* is 1 if surge BP or 0 if not. The functions of *f* and *g* are defined as:

1 $$ f=\prod \limits_{i=1}^ng\left({x}_i,{a}_i\right), $$

2 $$ g\left({x}_i,{a}_i\right)=\left\{\begin{array}{c}1\ \left(\mathrm{surge}\ \mathrm{BP}\right)\kern2em ,\kern0.5em {x}_i\ge {a}_i\ \\ {}0\ \left(\mathrm{not}\ \mathrm{surge}\ \mathrm{BP}\right)\kern0.75em ,{x}_i<{a}_i\ \end{array}\right., $$

where *x* = (*x*_1_,…, *x*_*n*_) is the selected features from input variables *x*' (all of features), *a* = (*a*_1_,…, *a*_*n*_) is the thresholds for *x*, and *n* is the number of the selected features. The *x* and *a* were selected and determined using categories of features known to CV medicine.

**Fig. 6:**
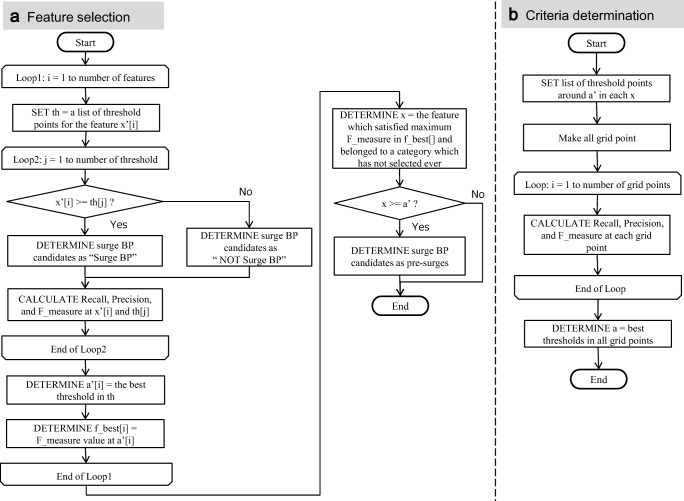
Algorithms of feature selection and criteria determination

In feature selection, *x* and *a*' (temporary thresholds) were determined as follows. Figure 6a shows the flowchart of feature selection.

First, each feature *x*_*i*_' was treated as a classifier by setting the thresholds. Then, three performance indexes (recall, precision, and F-measure [21]) were calculated for each threshold at each feature. The recall, precision, and F-measure are defined as (3), (4), and (5) respectively:

3 $$ \mathrm{Recall}=\frac{TP}{TP+ FN}, $$

4 $$ \mathrm{Precision}=\frac{TP}{TP+ FP}, $$

5 $$ \mathrm{F}-\mathrm{measure}=\frac{2\times \mathrm{Recall}\times \mathrm{Precision}}{\mathrm{Recall}+\mathrm{Precision}}, $$

where TP, FP, and FN indicate the number of true positives, the number of false positives, and the number of false negatives, respectively. The recall and precision are also known as sensitivity and positive predictive value, respectively. F-measure represents harmonic mean of the recall and precision. The *x*_*i*_ and *a*_*i*_' which had the highest F-measure were determined from each threshold in each feature and were temporarily formulated as function *g*. Surge BP candidates distinguished as “1” by the temporary *g* were defined as “pre-surges.”

Second, the above-mentioned process was repeated several times to “pre-surges” to help ensure exclusion of cases that were not actual surge BP cases. In the repeated process, another *x*_*i*_ and ${a}_i^{\prime }$ were selected from the remaining categories of features that had not been selected in previous processing. By applying this process to select *x*_*i*_ and *a*_*i*_′ from each category, the algorithm could cover the clinician’s viewpoints.

In criteria determination, values of *a* were determined by fine tuning *a*' with grid search. Figure 6b shows the flowchart of criteria determination. The determined *x* and *a* were formulated as final *g* and the classifier *f* was composed as the product of *g*. The cases determined as surge BP by the function *f* were treated as detected surge BP by this algorithm. The algorithm was developed with R using Rstudio.

### Performance evaluation

The performance of the algorithm was evaluated by following three viewpoints using data in high-reliability period, i.e., after data cleansing was finished. The surge BP classified by experts in the process of surge BP labeling was set as the target of reference for evaluation in 1 and 2, below.

1. Recall and precision: The mean values of recall and precision were calculated by using 5-fold cross validation to assess the generalization ability of the detection. These indexes (described as above) are also used as evaluation indexes. Study subjects were randomly assigned to five balanced groups by considering the frequency of surge BP of each group.
2. Mean absolute error between labeled surge BP and detected surge BP: To evaluate the detection accuracy of start points, we calculated the mean absolute error (MAE) [22] between time at start points of surge BP labeled by expert and those of detected surge BPs and the MAE between surge amplitudes of labeled surge BPs and those of detected surge BPs as:

6 $$ \mathrm{MAE}=\frac{1}{n}\sum \limits_{i=1}^n\left|{S}_{li}-{S}_{di}\right|, $$

where *n* is the number of surge BPs, *S*_*li*_ is an evaluation value of labeled surge BP, and *S*_*di*_ is an evaluation value of detected surge BP. The surge amplitude is defined as the difference between the SBP at the peak and the start points. The detection accuracy of start point of surge BP would reflect the quality of its detection because the start point is a foundation of the feature values. The detection accuracy of surge amplitude is important in clinical practice because it is regarded as a promising indicator of BPV, which reflects the additional risk factor of CV events.

3. Mean processing time to output the result: To evaluate the requirement of “high processing speed”, mean time of processing one measurement data point for study subjects was calculated for the following PC specifications that are common among clinicians in their clinical practice.

Number of CPU: 2

RAM: 7.5GB

CPU: Intel® Xeon®, 2.20GHz

## Results

Table 2 shows the clinical characteristics of the 94 study subjects. After the stage of “collection of surge BP labeled cases by CV experts”, a total of 3272 surge BPs were collected as so labeled from a total of 2,997,784 beats of continuous BbB BP data.

**Table 2:** Clinical characteristics and BP measured by oscillometric BP monitor

Characteristics	
Age	61.7 ± 11.6
Male:female	74:20
BMI, kg/m^2^	27.2 ± 5.2
Hypertension, %	64.8
Sleep apnea syndrome, %	33.0
Evening BP	
SBP, mmHg	131.3 ± 15.5
DBP, mmHg	78.0 ± 9.6
PR, beats/min	68.7 ± 9.6

*BMI* body mass index; *SBP* systolic blood pressure; *DBP* diastolic blood pressure; *PR* pulse rate. Data is expressed as mean ± standard deviation, number, or percentage. Evening BP values were measured by oscillometric BP monitor in supine position before starting BbB BP measurement.

During the development of the algorithm, the features were selected from four categories including upward, downward, reactivity phase, and recovery phase. Although the maximum number of selected features is six, we selected four features because the highest F-measure was at three or four. In case of the four features, the reactivity phase, which was important for clinical BP evaluation, [20] was selected; this was not included in three features. The features that reflect clinical significance, such as “recovery time,” were selected. All absolute values of correlation coefficients between the selected features were < 0.37, which suggests no multicollinearity.

**Fig. 7:**
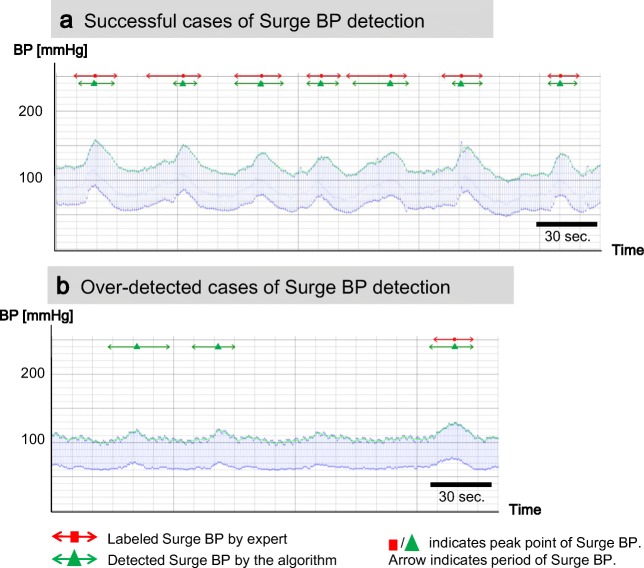
Typical cases of detected surge BP by the algorithm. **a** Successful cases. **b** Over-detected cases

Figure 7 shows typical cases of surge BP detection by the algorithm. Figure 7a presents a subject who had many surge BPs in a short period all of which were successfully surge BP detected. However, some start points detected by the algorithm differed from those of the labeled surge BPs. Figure 7b presents a subject who had overdetection of surge BP. Although there were three detected cases, two cases on the left side in the figure were not labeled by experts.

### Result of performance evaluation

**Fig. 8:**
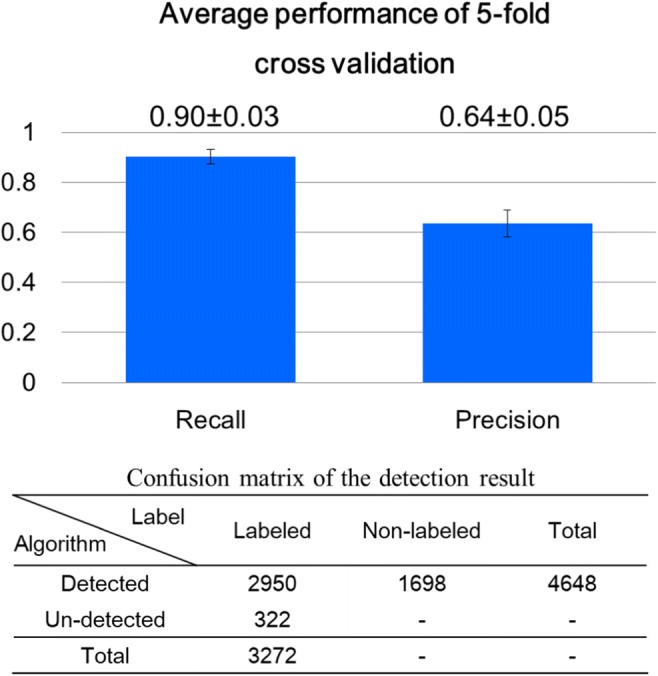
Performance of surge BP detection. Numeric values in each cell in confusion matrix are numbers of cases. The “-“indicates uncountable

The performance results for recall and precision by 5-fold cross validation are shown in Fig. 8. The mean value and standard deviation of recalls and of precisions in five groups were 0.90 ± 0.03 and 0.64 ± 0.05, respectively. The confusion matrix of detection result presents the number of surge BPs that were labeled or not by the expert (in columns) and the number of surge BPs detected or not by the algorithm (in rows), namely 2950 cases were successfully detected by the algorithm, 322 cases were not detected by the algorithm, and 1698 cases were overdetection cases.

**Fig. 9:**
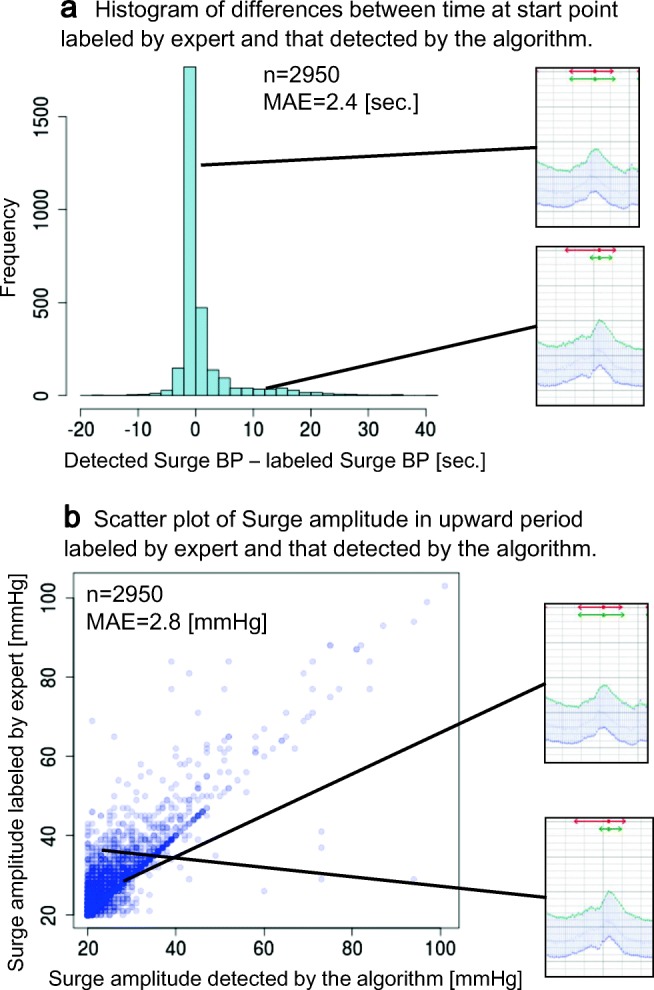
Performance of algorithm in detecting start point and calculation of surge amplitude in upward period

The accuracy of start points is shown in Fig. 9. Figure 9a shows a histogram of the time difference of the start points surge BP between labeled surge BP and detected surge BPs. Positive values on the horizontal axis mean that the detected start point is later than the labeled one. Most differences are distributed around 0 s, and the MAE was 2.4 s. Figure 9b is a scatterplot of surge amplitude of labeled surge BP vs. detected surge BPs. The MAE between surge amplitudes of labeled surge BP and detected surge BPs was 2.8 mmHg. Most of them were distributed on a line, which means no difference, or in the area above the line.

The mean value and standard deviation of processing time of the algorithm for 94 study subjects were 11.0 ± 4.7 sec.

### Comparison studies

Different classifiers that used the same features as our classifier were evaluated for comparison and are listed as follows: (1) linear discriminant analysis (LDA), (2) CART decision tree (C-DT), (3) logistic regression (LR), (4) support vector machine (SVM) using RBF kernel, and (5) AdaBoost (AB). The R packages used in LDA and LRA were {MASS}, C-DT was {rpart}, SVM was {e1071}, and AB was {adabag}.

**Fig. 10:**
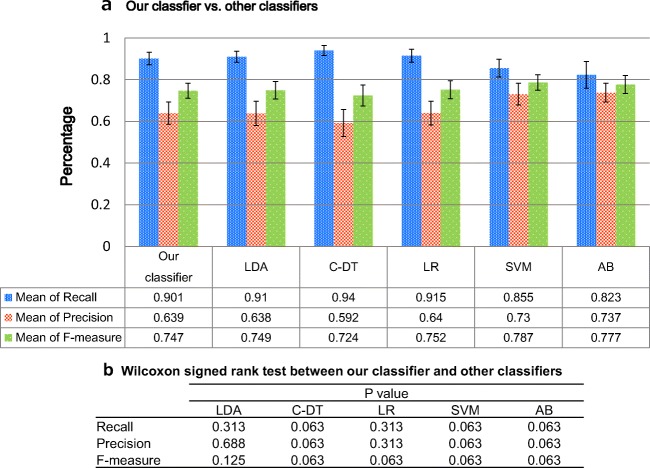
A comparison of our classifier vs. other classifiers. **a** Each bar graph indicates mean of recall, mean of precision, and mean of F-measure from left to right. These performance values were calculated through 5-fold cross validation. LDA: linear discriminant analysis; C-DT: CART decision tree; LR: logistic regression; SVM: support vector machine using RBF kernel; AB: AdaBoost are indicated. **b** The result of Wilcoxon signed rank test between our classifier and other classifiers

The hyper-parameters in C-DT, SVM, and AB were set to default values. These are representative methods for classifiers and include both linear and nonlinear models. The results of the comparative performance evaluation are shown in Fig. 10. There were no statistically significant differences between our classifier and the other classifiers, analyzed using the Wilcoxon signed rank test. We performed the tests using an R package {exactRankTests}.

## Discussion

We developed an automatic detection algorithm for surge BPs based on supervised learning using a BbB BP measurement device for the first time. This algorithm detected surge BPs by applying fixed criteria, and it satisfied the requirements of “high sensitivity of surge BP detection”, “understandability of detection rules”, and “high processing speed”. The details are described as below.

From the viewpoint of the high sensitivity requirement, our algorithm could detect surge BP with a high recall value of 0.90, which indicates that the algorithm is very useful to collect surge BP cases at an acceptable level for use in clinical practice, whereas clinicians needed to identify them visually in the past, which took a lot of time.

Precision was 0.64, which was within expectation considering the high sensitivity requirement for surge BP detection. As we showed in Fig. 7b, our algorithm detected non-labeled cases. There are several possibilities for this. First, these non-labeled but detected cases may be difficult for nonexpert staffs to identify surge BP because most of them occurred around surge BP thresholds. If the start point was identified as the very next beat by a nonexpert staff, some of these non-labeled cases detected by the algorithm satisfied the criteria of surge BP in that labeling phase. Second, human-error oversights of surge BPs by staffs would have occurred to some extent [23]. Smith [24] reported the error rate in routine tasks requiring careful work was over 1%. Because surge labeling requires more attention than routine tasks, the error rate by staffs could be even greater, especially when their stress or fatigue is cumulative. Third, the cases detected by the algorithm would include noisy BPVs. Further consideration is needed to exclude these cases from detection results from applying the algorithm. Although detected cases may include non-labeled cases due to reasons mentioned above, all detected cases will be used in future clinical studies. If more detailed features of surge BP that are associated with CVD risk are revealed in future clinical studies, the criteria of the algorithm could be improved to establish better standards for detecting surge BP.

Regarding the accuracy of detecting start points, which is a quite important function for determining the performance of the algorithm, most of them were detected correctly by the algorithm. However, some of those detected differed from those for the labeled surge BPs. In those cases, the detected start points tended to be closer to the peak than those in labeled surge BPs. This difference seems to have occurred due to a tendency that our algorithm detects a part of a surge BP that has a long upward period, and some surge BPs have two phases of increase in a single upward period (see lower case in Fig. 9a). (Upward period is the period between start point and peak point, and downward period is period between peak point and end point of surge BP candidates). Considering its mechanism, when sleep apnea occurs, the patient’s BP is gradually increased through the increase of sympathetic nerve activity induced by arterial oxygen desaturation [25]. After this phase of gradual BP increase, BP is rapidly and markedly increased at the time of release of sleep apnea; this is because the amount of venous return is increased during apnea due to increase of intrathoracic negative pressure [8]. Our algorithm tends to detect this rapid BP changing point as the start point.

The processing time of the algorithm was 11.0 ± 4.7 s, a relatively quick speed that is fit for actual medical consultation. It will not keep a clinician and a patient waiting for a long time to obtain an output of a surge report.

Finally, from the comparison studies, performance of our classifier was found to be as good as other classifiers; however, our classifier provides more explainability and understandability than comparative methods due to the clarity of each feature’s thresholds without clinician’s knowledge of classification model. To be an understandable algorithm for clinicians is quite important because the purpose of the algorithm is to assist establishing standard of surge BP in clinical study. Furthermore, only our classifier enables users to edit the threshold values for each feature independently. When useful features related to the mechanisms of BP surges become clear during future clinical studies, clinicians will be able to focus on and edit only the useful features.

Considering the mechanism of the BP variability is also important. The baroreflex is one of the most important physiological reflexes controlling arterial blood pressure [26]. The morning BP surge is one of the phenotypes of the BP variability closely determined by the sympathetic baroreflex [27]. In addition to surge BP, assessing the baroreflex sensitivity by customizing methods based on spontaneous variations [28, 29] is also necessary to reveal the mechanism of the variability.

Our study has limitations. First, it is not suitable for AF patients because they have large variations of BbB BP. This affects the detecting feature points and calculating feature values of surge BP. Second, we found in our previous study that the BP measured by the wrist-type device in the supine position tend to be approximately 5 mmHg higher than using the auscultation method at the upper arm in the supine position [30]. Therefore, the BbB BP values calibrated by the device in the present study have a potential error in the BP level.

## Conclusions

We developed an algorithm for automatic surge BP detection for clinical practice. The algorithm was developed by using supervised learning with labeled surge BPs. The classification model was very simple, and it enables the clinician to understand the detection rules. Although surge BPs were needed to be reviewed out manually in the past, a visual process that took a long time, this algorithm can correctly detect surge BPs with a recall of over 0.9. The result also suggests that the algorithm can detect surge BPs by using fixed criteria even if it is difficult for a staff to identify surge BP manually. Moreover, processing speed is enough to use in a typical medical consultation setting.

Clinicians would be able to see the patient’s summary report of nighttime surge BPs using our algorithm. The report provides the frequency of surge BPs and their peaks and amplitudes, which relate to risks of CV events. Understanding risks is important to both clinicians and patients in preventing CV events. Our automatic surge BP detection algorithm could contribute to clinical study on nocturnal hypertension, and it will be helpful to understand the mechanism of sleep onset of CVD events.

## Electronic supplementary material

ESM 1 (DOC 60 kb)
