# Supplementary material for: Automatic detection algorithm for establishing standard to identify “surge blood pressure”
Source: Med Biol Eng Comput. 2020 Apr 13;58(6):1393–404. doi: 10.1007/s11517-020-02162-4 (PMC7211788; doi:10.1007/s11517-020-02162-4)
Supplement: Supplementary file 1 — (DOC 60 kb) [file 11517_2020_2162_MOESM1_ESM.doc]

*Data cleansing*

The data cleansing was conducted by the members of study staff based on the following five abnormality criteria. First, measurements taken when the body is in motion should be excluded because motion can break the contact between the radial artery and tonometry sensor, thereby resulting in an inadequate BP measurement. The periods during which the body is in motion are determined using values from the triaxial-acceleration sensor. Second, the measurement device occasionally fails to detect the beat of the pulse properly. These periods, which can be attributed to temporary device error, should be regarded as low-reliability periods. Third, if any outlier values of BP are observed, they should be excluded due to low reliability. We determined outliers as systolic BP (SBP), diastolic BP (DBP), or pulse pressure (PP) above or below pre-set physiologically-based thresholds. Fourth, when subjects move their bodies, the contact between the radial artery and the sensor could change, occasionally resulting in large shifts in BP values. If BP values significantly and suddenly shift from the baseline level (under stable conditions before the BP shift) to extremely high or low levels, the period after the shift is not reliable and should be excluded. Fifth, the raw pressure signal from the sensor is weak, and BP values obtained during this time should be excluded because these periods are not reliable even though BP values are calibrated. The resulting periods retained after data cleansing were defined as high-reliability periods.

*Details of feature variables definition*

A framework for designing an activity recognition system of time-series data, which was proposed in a previous study [20], was modified and applied in this study. Whereas dividing raw time-series data into segments using a sliding window and calculating features for each segment were suggested in the previous study, for this study we added to the framework the function of detecting the feature points, which are very helpful for detecting Surge BP.

In peak point detection, the local maximum points were detected by using a sliding window, which has parameters of window size and shift size, and the maxima were treated as peak point candidates in Surge BPs.

In the start-end point detection algorithm, firstly, the start points in Surge BPs were detected as the final point during stable BbB BP in backward-searching ranges set before the peak point. Other parameters, including differences between each BbB BP value, were designed to detect the stable BbB BP periods. The threshold points were set for each parameter. Also, the start points were detected at each threshold point for each combination of parameters. The best-matched combination in terms of correspondence to the start point of labeled Surge BPs was adopted to parameters for start point detection. Secondly, the Surge BP end points were detected as the points which SBP had decreased by 75% of the surge amplitude from SBP at its peak in the forward-searching range. (The surge amplitude is the difference between SBP at peak point and that at start point.) The forward-searching range was set after the Surge BP peak point. Consequently, once the start point is detected, the end point is automatically detected.
